# Fabrication and Characterization of Chitosan-Polyethylene Glycol (Ch-Peg) Based Hydrogels and Evaluation of Their Potency in Rat Skin Wound Model

**DOI:** 10.1155/2021/4877344

**Published:** 2021-10-14

**Authors:** Md Mahmudul Hasan, Md Forhad Uddin, Nayera Zabin, Md Salman Shakil, Morshed Alam, Fahima Jahan Achal, Most. Hosney Ara Begum, Md Sakib Hossen, Md Ashraful Hasan, Md Mahbubul Morshed

**Affiliations:** ^1^Department of Biochemistry and Molecular Biology, Jahangirnagar University, Savar, Dhaka 1342, Bangladesh; ^2^Department of Biochemistry and Molecular Biology, Primeasia University, Banani, Dhaka 1213, Bangladesh; ^3^BCSIR Laboratories, Bangladesh Council for Scientific and Industrial Research, Shahbag, Dhaka 1000, Bangladesh

## Abstract

Thermal burns are a major cause of death and suffering around the globe. They can cause debilitating, life-altering injuries as well as lead to significant psychological and financial consequences. Several research works have been conducted in attempt to find a wound healing therapy that is successful. At present, hydrogels have been widely used in cutting-edge research for this purpose because they have suitable properties. This study aimed to see how therapy with chitosan-polyethylene glycol (Ch-Peg) based hydrogels affected the healing of burn wounds in rats. With the concern of public health, xanthan gum (X), boric acid (B), gelatin (Ge), polyethylene glycol (Peg), chitosan (Ch), glutaraldehyde (G), and HPLC-grade water were prepared using X : Ge : G, X : Ge : Peg : G, X : Ge : Ch : G, X : Ge : Peg : Ch : G, X : Ge : B : Ch : G, X : Ge : B : Peg : G, and X : Ge : B : Peg : Ch : G. The produced composite hydrogels were examined for swelling ability, biodegradability, rheological characteristics, and porosity. The 3D structure of the hydrogel was revealed by scanning electron microscopy (SEM). After that, the structural characterization technique named Fourier-transform infrared spectroscopy (FTIR) was used to describe the composites (SEM). Lastly, in a rat skin wound model, the efficacy of the produced hydrogels was studied. Swelling ability, biodegradability, rheological properties, and porosity were all demonstrated in composite hydrogels that contained over 90% water. Hydrogels with good polymeric networks and porosity were observed using SEM. The existence of bound water and free, intra- and intermolecule hydrogen-linked OH and NH in the hydrogels was confirmed using FTIR. In a secondary burned rat model, all hydrogels showed significant wound healing effectiveness when compared to controls. When compared to other composite hydrogels, wounds treated with X : Ge : Peg : Ch : G, X : Ge : B : Peg : G, and X : Ge : B : Peg : Ch:G recovered faster after 28 days. In conclusion, this research suggests that X : Ge : Peg : Ch : G, X : Ge : B : Peg : G, and X : Ge : B : Peg : Ch : G could be used to treat skin injuries in the clinic.

## 1. Introduction

Burns are injuries to the outer layer of the skin (i.e., epidermis) or other such living tissue induced by heat or other causative agents (radiation, electricity, chemicals), and can have a wide range of consequences [1]. The World Health Organization (WHO) claims that it is a global public health issue that causes an estimated 180,000 fatalities every year [2]. Burns have various effects on the tissue, organ, and system networks, including smoke inhalation, as well as a psychological influence [1, 3]. Despite the development of numerous antibacterial and antiseptic medications, scorch tissue damage remains a challenge for contemporary medicine [4]. Each year, about half a million Americans suffer from acute thermal injuries that necessitate medical attention, resulting in 40,000 hospitalizations and 3,400 deaths [5]. To date, substantial endeavor in burn treatments, such as the use of hydrogels as a wound dressing, have been designed to provide better the survival of patients.

Hydrogels are “three-dimensional, crosslinked networks of hydrophilic polymers” [6]. Because of their unique characteristics, including high sensitivity to physiological circumstances, hydrophilic attributes, stretchable tissue-like water holding capacity, and adequate flexibility, they are great candidates for biomedical applications [7, 8]. Furthermore, in recent times, composite hydrogels have gained more attraction as a competitive candidate for wound dressing materials due to their characteristics of biodegradability, biocompatibility, and ability to form a suitable environment for cell development [7, 9–11]. Among wound dressings, dressings cast from hydrogels, sometimes known as hydrocolloid dressings, have become the first major advancement and are used in drug delivery systems [12], biomedical applications [13], wound dressing [14], tissue engineering and regenerative medicine [15], biosensor [16], diagnostics [17, 18], and food additives [19, 20]. Currently, numerous wound dressing materials are available, including xanthan gum (X), gelatin (Ge), polyethylene glycol (Peg), and chitosan (Ch).

Xanthan is a polysaccharide-based polymer: “Xanthan gum (X) is a high-molecular-weight bacterial polymer produced by aerobic fermentation of *Xanthomonas campestris*” [21]. It has been used to immobilize enzymes and cells in hydrophilic matrix formulations for drug delivery applications [22]. It was utilized to make biodegradable skin scaffold hydrogels. The swelling behavior of X hydrogels was previously demonstrated by Bueno et al. [23]. Gelatin is a transparent, flavorless protein with the rheological feature of thermoreversible change from sol to gel [24]. It is widely used in the culinary, pharmaceutical, and photographic industries. According to several reports, gelatin is used for hemostasis in bleeding wounds [25].

Chitosan (Ch): “a linear polysaccharide composed of randomly distributed *β*-(1⟶4)-linked D-glucosamine and N-acetyl-D-glucosamine” containing some polar functionalities such as amino and -OH groups that make the polymer hydrophilic [26]. It attains a widespread application in drug delivery systems due to its biocompatibility, low toxicity, and biodegradability [27]. In medicine, it may be convenient in bandages to reduce bleeding and as an antimicrobial agent [28, 29]. In some studies, chitosan is widely used to make hydrogels for epidermal scaffolds [30, 31]. Chitosan-based hydrogels possess a higher swelling degree exhibiting smart hydrogels characteristics (“a smart hydrogel displays abrupt changes to its physical network nature as a response to external or internal stimuli”) [32]. Polyethylene glycol (Peg) is an ethylene oxide polymer that is generated when ethylene oxide comes into contact with ethylene glycol, ethylene glycol oligomers, or water [33]. It is a suitable element for biological applications because it does not usually express an immune response [34]. There have been many applications of polyethylene glycol identified ranging from industrial manufacturing to medicine [35]. It can be used for the preparation of hydrogels because of its biocompatibility and solubility in water [35]. PEG-hydrogels have been widely employed as drug delivery matrices and cell delivery vehicles to promote tissue regeneration [36]. PEG hydrogels with the appropriate design can help direct cellular processes such as survival, proliferation, secretion, and even differentiation [37, 38].

Fabrication of hybrid hydrogels is an exoteric strategy for improving biological characteristics. Even though chitosan and polyethylene glycol have several therapeutic properties, there is yet to study the production and characterization of chitosan-polyethylene-based hybrid hydrogels, as well as their wound-healing abilities. In this work, we prepared a number of hydrogels that emphasized on the usefulness of both chitosan and polyethylene glycol in combination, analyzed them, and investigated their efficacy in an Albino Wister rat skin wound model.

## 2. Materials and Methods

### 2.1. Materials

To produce Ch : Peg-based hydrogels, we used xanthan gum (X), boric acid (B), gelatin (Ge), polyethylene glycol (Peg), chitosan (Ch), glutaraldehyde (G), and HPLC-grade water. Sigma Aldrich (Darmstadt, Germany) provided B, Ge, Peg, Ch, and G, whilst Zhengzhou Sino Chemical Co. Ltd., provided X. (Zhengzhou, Henan, China). In the lab, HPLC-grade water was being produced. All raw materials were utilized in their natural state.

### 2.2. Synthesis of Hydrogels

To produce hydrogels, all components (X, B, Ge, Peg, Ch, and G) were dissolved in HPLC-grade water at different volume (Table 1). The mixture was then mixed until all of the components were dissolved, heated (85°C) to induce gel formation, and reweighed to assess evaporated water content from the sample mixture. The lost water volume was reintroduced into the mixture, and temperature was maintained at 85°C until the air-free gel formation. The gel was then placed into dried Petri dishes, allowed to cool at room temperature, and then stored at 4°C.

### 2.3. Examination of Moisture Content

The moisture content was calculated using the conventional IUPAC approach, with slight adjustments [8]. Firstly, 2 gm of samples was weighed in a porcelain crucible (which had been heated to 105°C, cooled, and weighed before). The sample was cooked for roughly two hours at 50°C in a temperature-controlled oven. The equation was used to determine the percentage of moisture in hydrogels:

(1) $$\begin{array}{c} \text{moisture content}\left(\%\right)=\left[\frac{\left(W_{1}-W_{2}\right)}{W_{1}}\right]\times 100, \end{array}$$

where *W*_1_ denotes the sample's original weight before drying and *W*_2_ denotes the sample's final weight.

### 2.4. Swelling Assessment of Prepared Hydrogels

The swelling was calculated following JIS K7223, a Japanese Industrial Standard. For 16 hours at room temperature, a dry gel (0.5 g) was submerged in HPLC water. [34]. After swelling, the hydrogel was filtered through a 100-mesh (149 m) stainless steel net. The following formula is used to determine swelling (*S*):

(2) $$\begin{array}{c} S=\frac{\left(m_{t}-m_{0}\right)}{m_{0}}, \end{array}$$

where the swollen gel's mass at time *t* is *m*_*t*_, while the dry gel's mass at time 0 is *m*_0_.

### 2.5. Time-Dependent Biodegradability Analysis

The hydrogels were dried to a consistent weight before first weighing to determine the biodegradability of prepared hydrogels. Hydrogels were then placed under the physiological solution for 7 to 21 days. After that, each hydrogel was taken and dried to a consistent weight once the stipulated duration was completed. The hydrogels were not placed into the physiological solution after weighing, and they were removed from the analysis [35].

### 2.6. Fourier Transform Infrared (FT-IR) Analysis

The materials were dried and pulverized before being mixed with KBr for FT-IR analysis. The FT-IR- 8400S spectrometer was used to capture the FT-IR spectrum (Shimadzu, Japan). The range remains between 600 and 4000 cm^−1^.

### 2.7. Hdrogel's Morphology Assessment by Scanning Electron Microscopy (SEM)

To perform SEM study, the interior structure of the dehydrated hydrogels was exposed by cutting them apart. The hydrogel's morphology was then examined to confirm that it retained its structure. A 15 kV working voltage was utilized using a HITACHI S3400 N SEM (Johnson County, Iowa, United States).

### 2.8. In Vivo Wound Healing Potential of Synthesized Hydrogels

**Figure 1:**
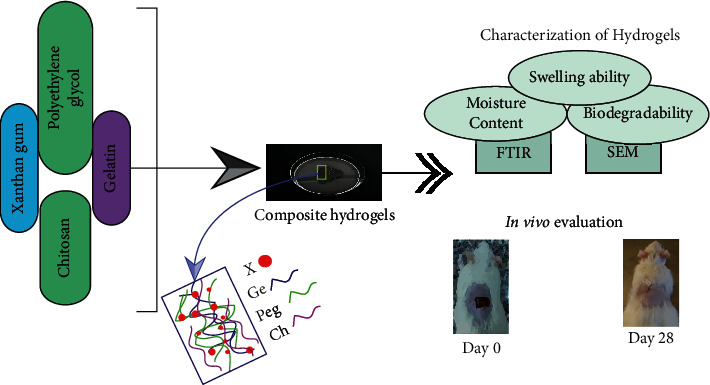
An overview of the present research.

In vivo wound healing was investigated on adult Albino Wister rats, 5-6 weeks of age and about 160 to 170 grams weight, which were housed in plastic cages for one week to be adapted to the measurement conditions. The experiments involved ten groups, each with four rats, and the study lasted 28 days (Figure 1). It was strictly confirmed that rats experienced a natural day-night cycle regularly. Standard laboratory diet and sterile water under normal conditions (temperature/humidity) were provided. The research was carried out in accordance with the “Bangladesh Association for Laboratory Animal Science's ethical guidelines.”

The method for creating burn wounds we followed a process is as follows: 70% IPA was used for disinfectant purposes after shaving the skin of the rats. Then, in rats under moderate ketamine anesthesia, a cylindrical metal rod (12 mm diameter) was heated for 1 minute in boiling water at 95°–100°C and pushed to the shaved surface of the skin [22]. The rats in groups 3 to 10 were treated by dressing them with the prepared composite hydrogels, while group 1 was left untreated as a negative control. As a positive control, rats in group 2 were given povidone-iodine 5% ointment manufactured by Jayson Pharmaceuticals Ltd. [11].

### 2.9. Wound Area Measurement

After applying hydrogels (0.1 M/mL) to the wound site on a daily basis at a set time, we inspected the wounds of each group for 7, 14, 21, and 28 days, and the wound area was measured by tracing the wound boundaries on a transparent paper before images were taken (Huawei Y7, camera specification: 12 MP, f/2.2, 1/2.9″, 1.25 *µ*m, PDAF). A graph paper was used to record the wound areas of all groups, and the authors who took the data were blind about the treatment. The size of the wound was represented as mm^2^ of its original size.

### 2.10. Statistical Analysis

The data were initially organized using Microsoft Excel 2016 (Redmond, Washington, USA). Then, using GraphPad Prism, the data were submitted to a two-way analysis of variance (ANOVA), and the differences between groups were examined using the Bonferroni post hoc test (version 8.0.2, San Diego, California). Results are displayed as the mean ± standard deviation (SD). The differences were judged significant at *p* value <0.05.

## 3. Results and Discussion

### 3.1. Moisture Content Assessment

**Figure 2:**
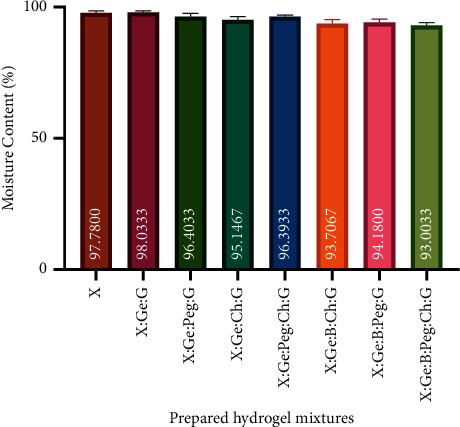
Moisture content of prepared hydrogel mixtures. All hydrogels had a water content of more than 93%. This strongly shows that hydrogels are effective at donating water to wound sites and that the water content of hydrogels helps wound healing by keeping the environment wet. Results are expressed as mean ± SD. *Abbreviations.* Xanthan gum (X), boric acid (B), gelatin (Ge), polyethylene glycol (Peg), chitosan (Ch), and glutaraldehyde (G).

Hydrogels can hold a large amount of water while maintaining their dimensional stability [39]. Moisture content, on the other hand, is fundamental for hydrogel integrity and naturalizes solubility and diffusion of chemicals, both of which are critical for therapeutic systems [40]. Furthermore, the moisture content of hydrogels can change the volume of the hydrogel, which is controlled by external variables such as type of solvent, ionic strength, and temperature [41].  Figure 2 shows the moisture content values of the synthesized hydrogels, indicating that hydrogels are effective at donating water to the wound site and that hydrogels' water content supports wound healing by maintaining a moist environment. In this experiment, X was a major component and it includes hydrophilic-like groups such as -CHO, -OH, and -COOH. Other components used in hybrid hydrogels include hydrophilic groups in their structure, such as Ge, which has -COO and -NH2 hydrophilic groups, and G, which has -CHO and -COO hydrophilic groups. Thus, the hydrophilic groups present in these hydrogels strongly maintain the moisture content (about 93 to 98%) of various hybrid composite hydrogels. Shawan et al. reported that X, citric acid (CA), Ge, or G containing hydrogel carried over 90% of moisture content [22].

### 3.2. Time-Dependent Swelling Ability of Hydrogels

**Figure 3:**
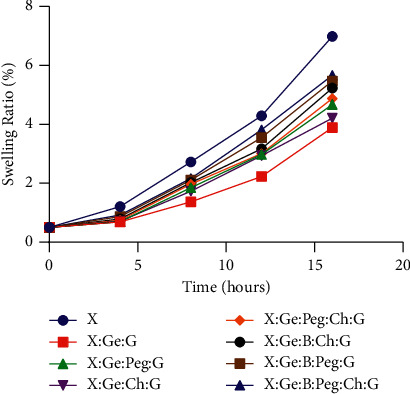
Swelling ability of hydrogel composites. After 16 hours, the swelling ratio of all hydrogels ranged from 3.89 to 6.98, confirming their suitability for use as a wound dressing. A swelling ratio of 6.98 was observed in hydrogels made only from (X). When gelatin and glutaraldehyde were used in the mixture, the swelling ratio was drastically lowered to 3.89. (i.e., X : Ge : G). *Abbreviations.* Xanthan gum (X), boric acid (B), gelatin (Ge), polyethylene glycol (Peg), chitosan (Ch), and glutaraldehyde (G).

The tendency of hydrogels to swell is critical in wound dressings because it influences the wound's potential to withstand a specific amount of exudate and maintain a humid environment at the wound site [42, 43].  Figure 3 illustrates that the swelling ratio of all hydrogels varied from 3.89 to 6.98 after 16 hours, indicating that they are suitable for topical treatment [44]. The swelling ratio of hydrogels manufactured from X was 6.98. The swelling ratio was dramatically reduced to 3.89 when gelatin and glutaraldehyde were added in the formulation (i.e., X : Ge : G). It is noted that the swelling capacity is inversely proportional to the crosslinking density and it expresses the degree of hydrophilicity of the hydrogels [45]. Previously, it has been reported that pure X chains possess a measurable number of groups that can be cross-linked [45]. A prior study reported that Ge facilitates cross-linking in the X [45]. When Peg (10 mg) and Ch (20 mg) were added to the existing gel separately to form two different samples, the swelling capacity was 4.67 for X : Ge : Peg : G and 4.21 for X : Ge : Ch : G which was nearly equal. Similarly, X : Ge : Peg : Ch : G hybrid hydrogel has a swelling ratio of 4.88. Subsequently, when X was interacted with B in the formulation (i.e., X : Ge : B : Ch : G, X : Ge : B : Peg : G, and X : Ge : B : Peg : Ch : G), the formulation's capacity to swell rose somewhat from 5.23 to 5.66. The hydrophilic groups contained in the gel likely triggered the slight improvement in swelling capacity of these hybrid hydrogels. The finding suggests that, as the concentration of gel raised, the cross-linking density may rise [46].

### 3.3. Time-Dependent Biodegradability Assessment

**Figure 4:**
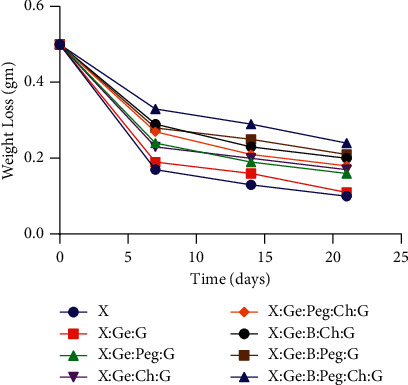
Biodegradability of hydrogel composites. After 21 days, all of the fabricated hydrogels showed biodegradability, with weights ranging from 0.1 to 0.24 of their initial weight. Notably, all composite hydrogels were made from 0.5 g of material, and the hydrogels made from solely X had the best biodegradability. When compared to simply X hybrid hydrogels, the presence of Ge and G did not affect the degrading behavior (0.11 versus 0.10 of their initial weights). When chitosan and polyethylene glycol were combined with boric acid, the composite hydrogels (i.e., X : Ge : B : Ch : G, X : Ge : B : Peg : G, and X : Ge : B : Peg : Ch : G) showed a tendency to degrade less, possibly due to cross-linking density. *Abbreviations*. Xanthan gum (X), boric acid (B), gelatin (Ge), polyethylene glycol (Peg), chitosan (Ch), and glutaraldehyde (G).

Biodegradability is one of the major properties of hydrogels because it promotes tissue regeneration, and so it is directly involved in the wound healing process [47]. In our study, we assessed the degradation characteristics of the composite hydrogels by measuring the effect of polymer composition on biodegradability. It is clear from Figure 4 that the disintegration of the hydrogels decreased as the polymer content increased, and all produced hydrogels showed biodegradability after 21 days, with weights ranging from 0.1 to 0.24 of their initial weight. Notably, the composite hydrogels were taken initially as 0.5 g, and the hydrogel prepared from X showed the highest biodegradability. In comparison with previous hybrid hydrogels, the addition of other materials in the hydrogel synthesis resulted in a reduction of degradation, for example, the X : Ge : B : Peg : Ch : G hybrid hydrogel had a minimal degradability of 0.24. Mechanical fragility and quicker breakdown trend are two drawbacks of Ch-Peg-based hydrogels [48]. Avoiding the drawbacks, beneficial properties can be used in the composition. The presence of Ge and G did not change the degradation behavior compared to X hybrid hydrogels (0.11 versus 0.10 of their initial weight). However, when chitosan and polyethylene glycol were added with boric acid, the composite hydrogels (i.e., X : Ge : B : Ch : G, X : Ge : B : Peg : G and X : Ge : B : Peg : Ch : G) showed a tendency to decrease the degradation, which might happen due to cross-linking density [44].

### 3.4. Morphological Characterization of Prepared Hydrogels

**Figure 5:**
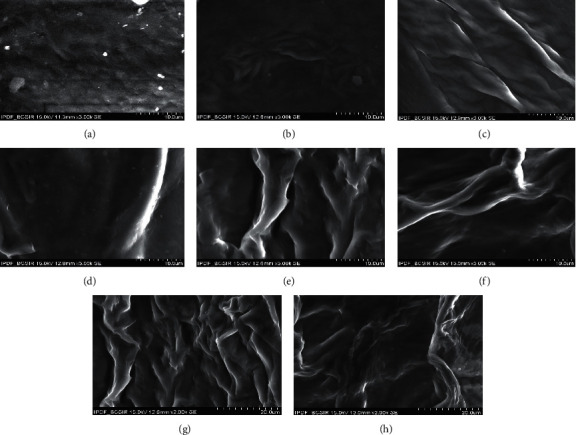
Microscopic surface appearance of the produced hydrogels. All of the hydrogels exposed well-developed cross-linked networks and porous topology. (a) X hydrogel surface morphology, (b) X : Ge : G surface morphology, (c) X : Ge : Peg : G surface morphology, (d) X : Ge : Ch : G surface morphology, (e) X : Ge : Peg : Ch : G surface morphology, (f) X : Ge : B : Ch : G surface morphology, (g) X : Ge : B : Peg : G surface morphology, and (h) X : Ge : B : Peg : Ch : G surface morphology. *Abbreviations.* Xanthan gum (X), boric acid (B), gelatin (Ge), polyethylene glycol (Peg), chitosan (Ch), and glutaraldehyde (G).

SEM is suitable for studying surface morphology and hydrogel porosity [49].  Figure 5 illustrates the morphology of the hydrogel conformations. All hydrogels showed quite well cross-linked networks and porous topology. The hydrogel surface is mostly homogeneous, looking smooth and thick, and porous structure has a significant impact on nutrition and oxygen transmission, exclusively in the dearth of a functioning vascular system. As a consequence, our hydrogels' porous nature reveals their beneficial influence on wound healing. [50].

### 3.5. Fourier Transform Infrared Spectroscopy (FT-IR) Analysis

**Figure 6:**
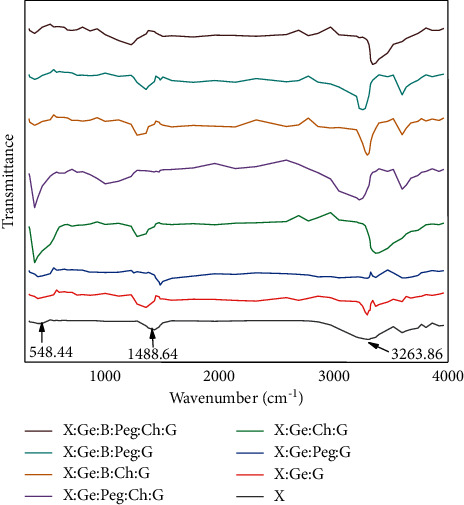
Hydrogel composites' FTIR spectra. The stretching vibration of -COO ester bonds, C-N stretching, and OH bending cause peaks in the spectra between 1404 and 1500 cm^−1^. Because of the (N-H) group and hydrogen-bonded hydroxyl group (O-H), a wide band formed at 3220–3334 cm^−1^. *Abbreviations.* Xanthan gum (X), boric acid (B), gelatin (Ge), polyethylene glycol (Peg), chitosan (Ch), and glutaraldehyde (G).

“FTIR (Fourier transform infrared spectroscopy)” is a tool for identifying and characterizing unknown materials, detecting impurities in a substance, detecting additives, and determining breakdown and oxidation [44, 51]. The FT-IR spectroscopy of the produced samples is interpreted in Figure 6 and Figure S2. Peaks between 1404 and 1500 cm^−1^ in the spectra are attributable to the stretching vibration of -COO of ester bonds, C-N stretching, and OH bending [44, 52]. The hydrogen-bonded hydroxyl group (O-H) and amino group (N-H) produced a wide band at 3220–3334 cm^−1^, which indicated stretching vibration of H-bonded NH and free, intra-, and intermolecule bound hydroxyl group [53]. At peaks in the range of 1400 to 100 cm^−1^, the “C-O stretching strong modes” revealed their features [54]. Between the two spectra, there are noticeably multiple peaks. These spectra have remarkably similar absorption peaks in the 1000–500 cm^−1^ region. Signature pick refers to peaks that occur at 400–600 cm^−1^ [55].

### 3.6. Wound Contraction Rate in Albino Wistar Rat

**Figure 7:**
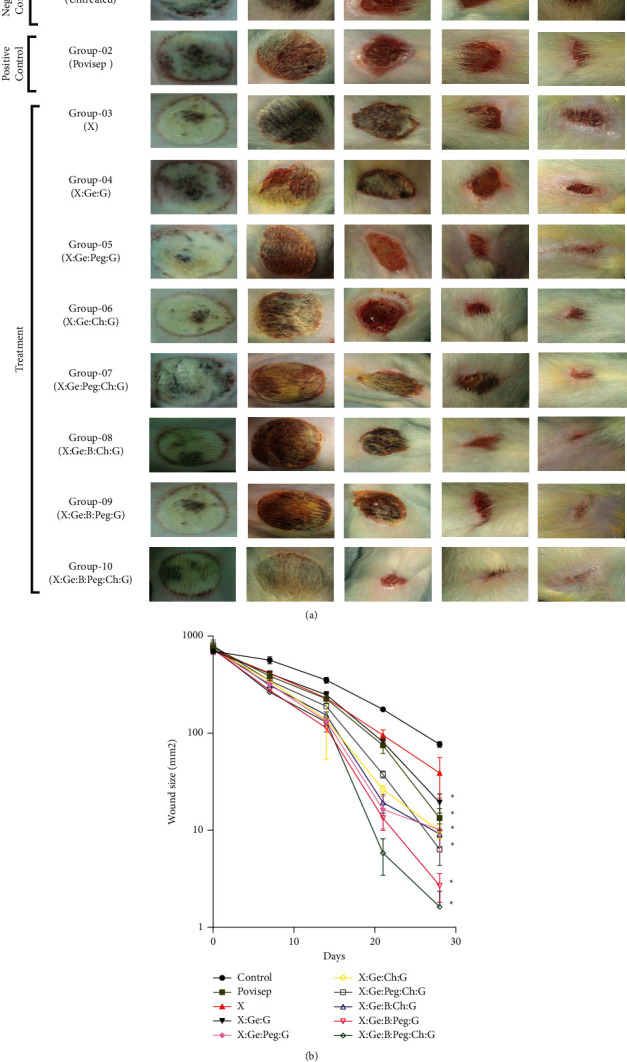
(a) Improvement of wound healing using hydrogel composites in an experimental second-degree burn of rat skin. Wounds treated with X : Ge : Peg : Ch : G, X : Ge : B : Peg : G, and X : Ge : B : Peg : Ch:G recovered the most after 28 days when compared to other hydrogel compositions. The presence of Peg and Ch in the formulations may explain for the hydrogel composites considerable wound contraction. *Abbreviations.* Xanthan gum (X), boric acid (B), gelatin (Ge), polyethylene glycol (Peg), chitosan (Ch), and glutaraldehyde (G). (b) Diagram of wound healing contraction rate. At the end of 28 days treatment period wound area of 28th day was analyzed using a two-way ANOVA coupled with Bonferroni posthoc test. ^*∗*^Significantly different from control, none of the hydrogel treated groups were significantly different from the povisep group (*p* < 0.05). *Abbreviations.* Xanthan gum (X), boric acid (B), gelatin (Ge), polyethylene glycol (Peg), chitosan (Ch), and glutaraldehyde (G).

Figure 7 shows that, after seven days of using hydrogels and povisep, the skin of the negative control, positive control (povisep), and treatment groups was hemorrhagic, with no evidence of infection or wound contraction. It is apparent from Figures 7(a) and 7(b) that, after 28 days of hydrogel application, all the hybrid hydrogel-treated injuries displayed greater wound closure rate compared to negative controls except X. However, none of the hydrogel-treated groups were significantly different from the povisep group (*p* < 0.05) (Figure 7(b) and Figure S1). Several studies have previously demonstrated that wound epithelialization occurs more quickly when the wound environment is kept wet [56]. This could be due to facile movement of keratinocytes over an injured moist layer [56]. It is easily evident from Figures 2 and 3 that all of the composite hydrogels retain more than 93 percent of their weight in water and exhibit exceptional swelling properties. As a result, it is conceivable that they' will be able to keep the wound wet. Markedly, after applying composite hydrogels for 28 days, X : Ge : Peg : Ch : G, X : Ge : B : Peg : G, and X : Ge : B : Peg : Ch : G treated wounds exhibited higher recovery as opposed to other composite hydrogels (Figures 7(a) and 7(b)). Previously, Shawan et al. showed that X : Ge : G, and X : CA : Ge : G showed higher contraction rate compared to X, X : CA, X : Ge : G or X : CA : Ge : G hydrogel after 20 days [22]. Results indicate that CA conjugation in X : Ge : G had no distinguishable effects on wound contraction rate. As no positive control was included in the study of Shawan et al. and statistical significance was not reported in response to the negative control, the effects of the hydrogels itself could not determine from this study [22]. In the current study, none of the synthesized hydrogels were significantly different from the positive control (povisep group) after 28 days, and this might be due to higher variation of contraction rate in this group. However, the higher wound contraction was seen in Peg or Ch-containing hydrogels. Peg or Ch has some beneficial role in tissue regeneration because they promote various cellular functions, for example, migration, adhesion, and proliferation [15, 57–60]. Furthermore, Peg, B, and Ch have an antimicrobial activity that reduces chances of infection and improves healing in the wound area [61–63]. Thus, it can be said that if the stability of Peg, B, or Ch maintains perfectly, in comparison with other hybrid composite hydrogels, these three composite hydrogels (X : Ge : Peg : Ch : G, X : Ge : B : Peg : G, and X : Ge : B : Peg : Ch : G) can largely perform in terms of possible wound healing.

## 4. Conclusion

In this study, X, B, Ge, Peg, Ch, G, and ultrapure water were used to construct a group of hybrid biomaterials or hydrogels, i.e., X, X : Ge : G, X : Ge : Peg : G X : Ge : Ch : G, X : Ge : Peg : Ch : G, X : Ge : B : Ch : G, X : Ge : B : Peg : G, and X : Ge : B : Peg : Ch : G. All of the produced hybrid hydrogels had a moisture content of more than 93%, with swelling abilities ranging from 3.89 to 6.98 after 16 hours. After 21 days, all of the hydrogels were found to be biodegradable, with weights ranging from 0.1 to 0.24 of their original weight. The existence of bound water as well as free, intra- and intermolecule hydrogen linked OH and NH was confirmed by FT-IR investigations. The hydrogels had good polymeric networks and porosity, according to scanning electron microscopy. All of the hydrogels were effective at wound healing. Compared to other hydrogel compositions, wounds treated with X : Ge : Peg : Ch : G, X : Ge : B : Peg : G, and X : Ge : B : Peg : Ch : G recovered the most after 28 days. The presence of Peg and Ch in the formulations may account for the significant wound contraction of these hydrogel composites. Finally, this study confidently state that X : Ge : Peg : Ch : G, X : Ge : B : Peg : G, and X : Ge : B : Peg : Ch : G are potential wound dressing materials.

## Figures and Tables

**Table 1:** An overview of hydrogel formulation.

Hydrogel sample	Nomenclature	Xanthan gum	Gelatin	Boric acid	Polyethylene glycol	Chitosan	Glutaraldehyde	HPLC water
		(mg)	(mg)	(mg)	(mg)	(mg)	(ml)	(ml)
1	X	600	—	—	—	—	—	100
2	X : Ge : G	600	3	—	—	—	0.5	99.5
3	X : Ge : Peg : G	600	3	—	10	—	0.5	99.5
4	X : Ge : Ch : G	600	3	—	—	20	0.5	99.5
5	X : Ge : Peg : Ch : G	600	3	—	10	20	0.5	99.5
6	X : Ge : B : Ch : G	600	3	3	—	20	0.5	99.5
7	X : Ge : B : Peg : G	600	3	3	10	—	0.5	99.5
8	X : Ge : B : Peg : Ch : G	600	3	3	10	20	0.5	99.5

*Abbreviations.* Xanthan gum (X), gelatin (Ge), Boric acid (B), polyethylene glycol (Peg), chitosan (Ch), glutaraldehyde (G).

## Data Availability

A data access committee will make data available upon request (Md. Mahmudul Hasan: mahmud.bmbju@gmail.com; Nayera Zabin: zabinnayera@yahoo.com; Md. Salman Shakil: salman.shakil@primeasia.edu.bd; Mahbubul Morshed: mahbubul.morshed@juniv.edu).

## Supplementary Materials

Supplementary Materials Figure S1: graphical abstract. Figure S2: FTIR spectra of hydrogel composites. (A) X hydrogel, (B) X : Ge : G hydrogel, (C) X : Ge : Peg : G hydrogel, (D) X : Ge : Ch : G hydrogel, (E) X : Ge : Peg : Ch : G hydrogel, (F) X : Ge : B : Ch : G hydrogel, (G) X : Ge : B : Peg : G hydrogel, and (H) X : Ge : B : Peg : Ch : G. *Abbreviations.* Xanthan gum (X), boric acid (B), gelatin (Ge), polyethylene glycol (Peg), chitosan (Ch), and glutaraldehyde (G).
