# Supplementary material for: Fabrication and Characterization of Chitosan-Polyethylene Glycol (Ch-Peg) Based Hydrogels and Evaluation of Their Potency in Rat Skin Wound Model
Source: Int J Biomater. 2021 Oct 14;2021:4877344. doi: 10.1155/2021/4877344 (PMC8531824; doi:10.1155/2021/4877344)
Supplement: Supplementary Materials — Figure S1: graphical abstract. Figure S2: FTIR spectra of hydrogel composites. (A) X hydrogel, (B) X : Ge : G hydrogel, (C) X : Ge : Peg : G hydrogel, (D) X : Ge : Ch : G hydrogel, (E) X : Ge : Peg : Ch : G hydrogel, (F) X : Ge : B : Ch : G hydrogel, (G) X : Ge : B : Peg : G hydrogel, and (H) X : Ge : B : Peg : Ch : G. Abbreviations. Xanthan gum (X), boric acid (B), gelatin (Ge), polyethylene glycol (Peg), chitosan (Ch), and glutaraldehyde (G). [file 4877344.f1.docx]

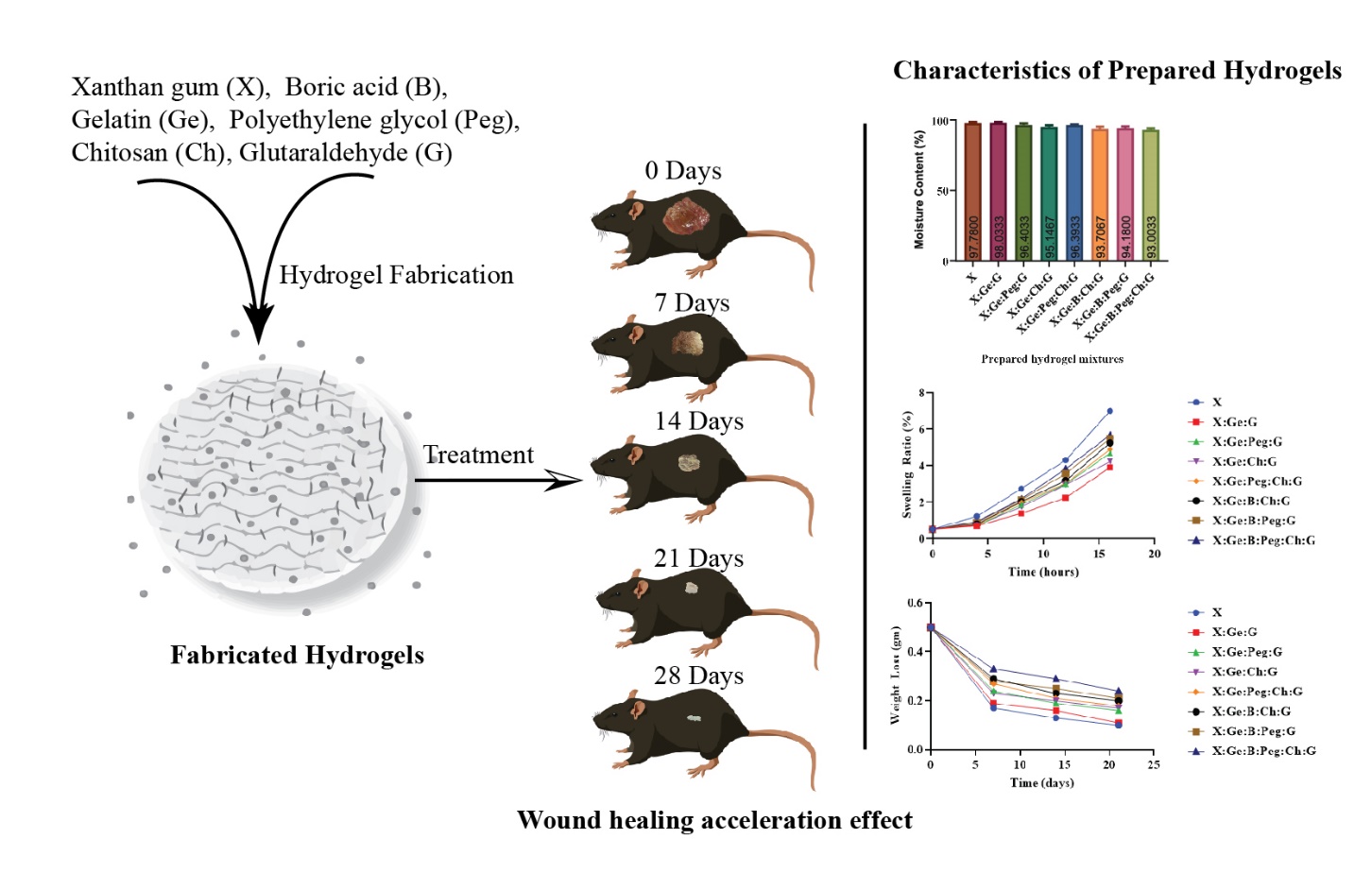


**Figure S1:** Graphical abstract.


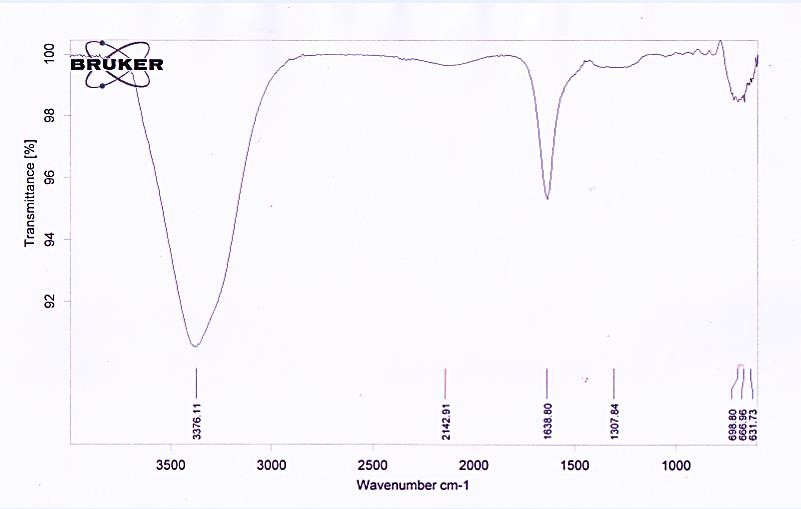


(A)


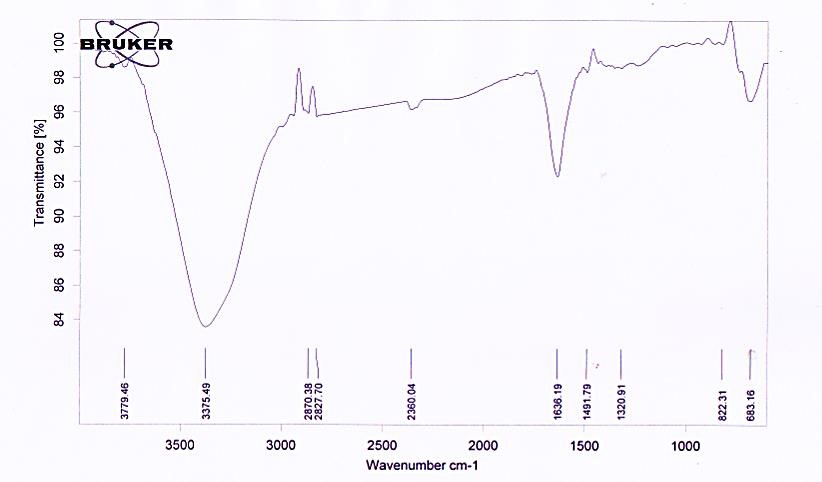


(B)


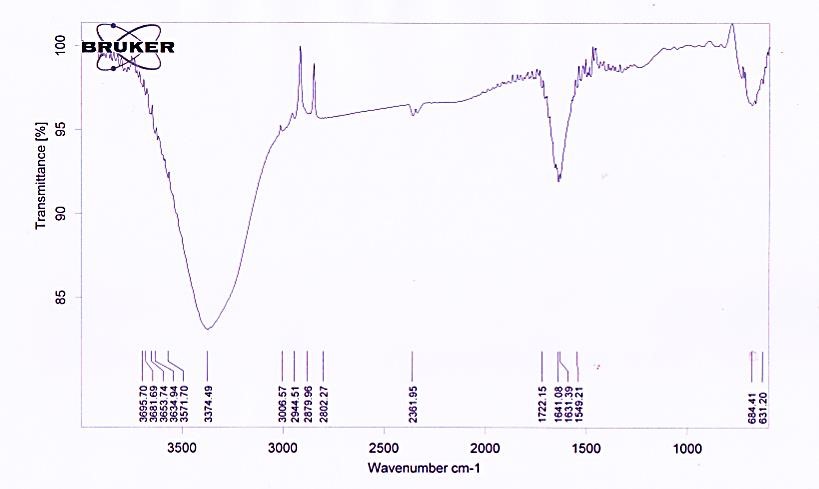


(C)


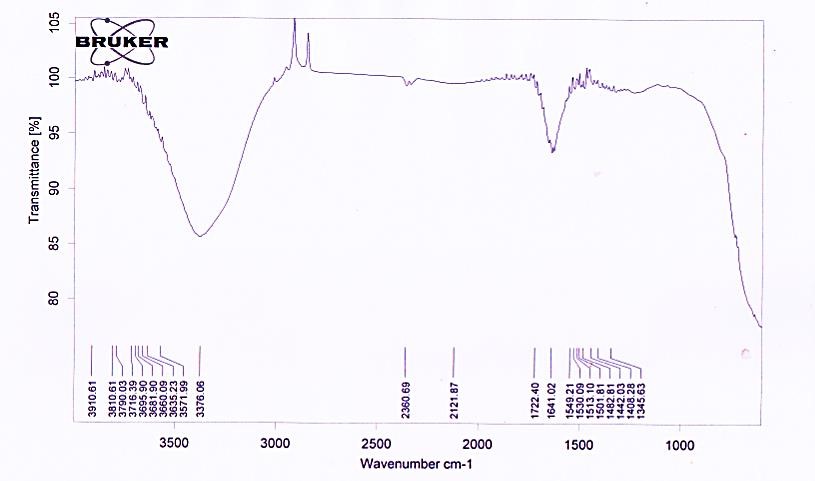


(D)


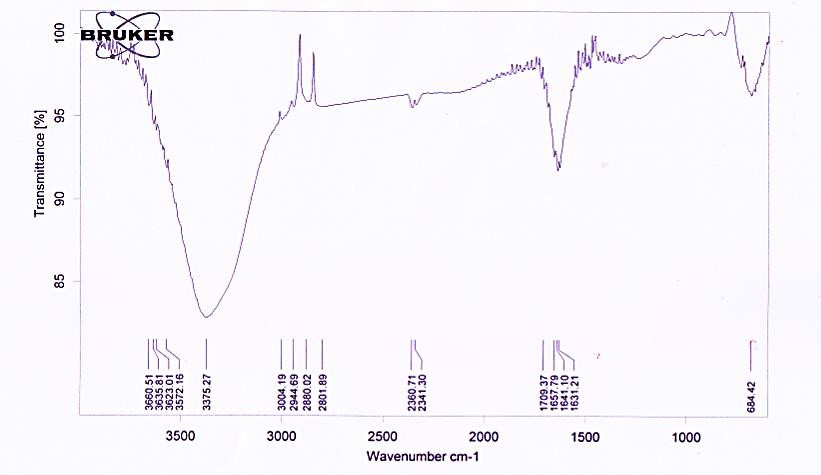


(E)

(F)


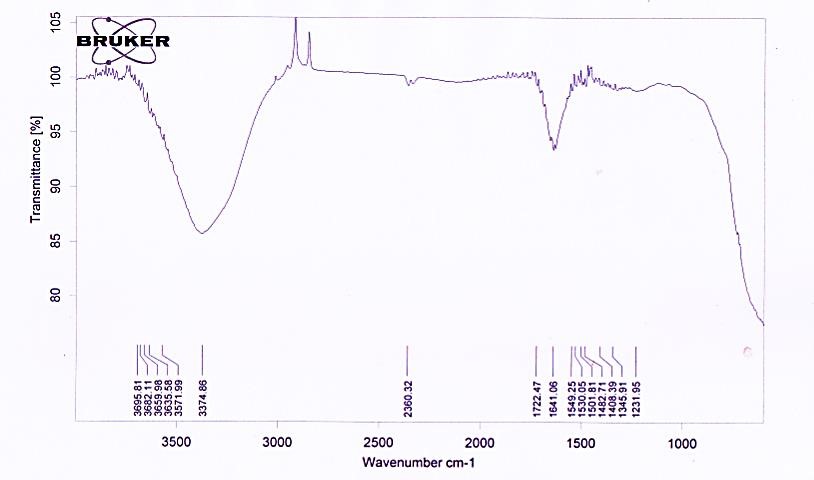


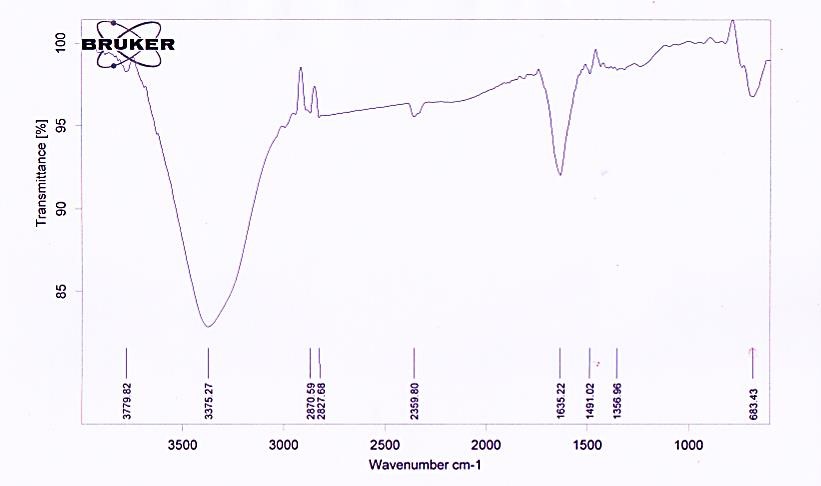


(G)


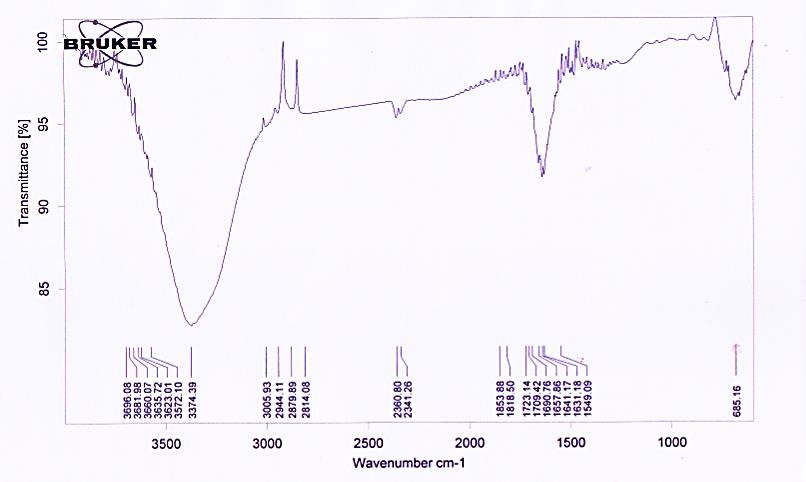


(H)

**Figure S2:** FTIR spectra of hydrogel composites. (A) X hydrogel hydrogel, (B) X: Ge: G hydrogel, (C) X:Ge:Peg:G hydrogel, (D) X:Ge:Ch:G hydrogel, (E) X:Ge:Peg:Ch:G hydrogel, (F) X:Ge:B:Ch:G hydrogel, (G) X:Ge:B:Peg:G hydrogel, and (H) X:Ge:B:Peg:Ch:G

**Abbreviations:** Xanthan gum (X), boric acid (B), gelatin (Ge), polyethylene glycol (Peg), chitosan (Ch), glutaraldehyde (G).
